# The opportunities of research parasitism: A case study using the Barcode of Life Data System (BOLD)

**DOI:** 10.1093/gigascience/giac123

**Published:** 2022-12-06

**Authors:** Jack Pilgrim

**Affiliations:** Institute of Infection, Veterinary and Ecological Sciences, University of Liverpool, Liverpool, L69 3BX, UK

**Keywords:** research parasitism, case study, BOLD, barcoding, *Rickettsia*, symbiosis, endosymbionts

## Abstract

The Barcode of Life Data System (BOLD) is primarily used to identify biological specimens based on a mitochondrial gene sequence and has been an underpinning resource for life science researchers. Importantly, curators of BOLD archive DNA extracts where possible, and also record contaminant sequences that can be made available on request. This collegial offering of samples and data led to our work describing the serendipitous discovery of new interactions between a Torix *Rickettsia* bacterium and their arthropod hosts and resulted in winning the 2022 Junior Research Parasite Award. A case study of this work is presented, which discusses the opportunities provided by secondary data and how careful maintenance of such large-scale repositories plays a vital role in scientific research that goes beyond obvious lines of enquiry.

## Background

Recently, in recognition of rigorous secondary analysis, I was awarded the 2022 Junior Parasite award for a paper I led describing the discovery of *Rickettsia* amplicons in the Barcode of Life Data System (BOLD) [1]. *Rickettsia*, a genus of bacteria best known as the causative agent of typhus, contains a nonpathogenic subgroup (Torix; otherwise known as *Tisiphia*sp. [2]), which, until our study, was poorly characterized in terms of its genetic diversity or host range. Our scouring of BOLD was supplemented by a search of whole-genome projects from the Sequence Read Archive (SRA) and a targeted screen that allowed for the discovery of multiple new Torix–arthropod associations, including in parasitoid wasps, spiders, and insect vectors (such as mosquitoes and black flies). Considering similar intracellular (endosymbiotic) bacteria are currently being studied in vector-borne disease control initiatives, the latter observation opens a key avenue of research as to whether Torix *Rickettsia* can alter vectorial capacity. Below I reflect on lessons learned during the completion of this piece of work.

## The lifeline of research parasitism

Prior to the big data revolution, researchers’ only option was to struggle through acquiring primary data for their respective projects. Through no fault of their own, investigators have often been thwarted by negative results, difficulties in establishing and maintaining necessary resources, and financial constraints. As a PhD student, I encountered such barriers. My project for the 4 years of doctoral training involved understanding the potential of endosymbionts to affect the vectorial capacity of *Culicoides* biting midges, which transmit several pathogens of animals and human health relevance. The most famous application for insect vector–endosymbiont systems is *Wolbachia*-infected mosquitoes, which, when released into the wild, can induce a viral inhibitory effect to reduce the health impact of pathogens such as dengue virus [3]. The hope was to expand this innovation to neglected insect vectors such as *Culicoides*. Although a particular species (*Culicoides pulicaris*) was initially considered an ideal candidate for such work prior to commencing my PhD, failure to replicate this preliminary work meant that other avenues of investigation were needed. Our subsequent discovery of a novel and widespread midge endosymbiont, a Torix *Rickettsia* [4], meant new lines of enquiry were possible but difficult. Infection of a cell line could take months to years, colonized species available in the United Kingdom (of which only 2 exist) were not infected, and field-caught insects were only available a few months of the year. These difficulties of establishing a tractable *Rickettsia*-midge system left me with limited options [5].

It was at this point that research parasitism threw me a lifeline. The multilocus *Rickettsia* designation system I had devised at the beginning of the project to screen midges contained *COI*, a gene commonly used in mitochondrial DNA barcoding, a technique to identify existing species and aid in the discovery of new species. Surprisingly, when *Rickettsia COI* sequences were searched for matches on NCBI, several hits represented deposits that have been recorded as of insect origin—implying that classic barcoding approaches were also accidentally amplifying the *COI* gene from *Rickettsia* symbionts of the insects.

This led me to examine the BOLD database [6]—a massive repository for *COI* sequences from eukaryotic DNA barcoding projects. When comparing my Torix *COI* sequences to the BOLD database, several hundred entries were returned. On first viewing, it appeared surprising that amplicons originating from bacteria would be found in a database designed to store mitochondrial sequences. However, the few bacteria likely to share homology with mitochondria are the Rickettsiales order of alphaproteobacteria (containing the genera *Wolbachia* and *Rickettsia*, amongst others), where the hypothetical bacterial ancestor of mitochondria (proto-mitochondria) is thought to be phylogenetically embedded. These observations indicated that inadvertent *Rickettsia* amplification was widespread and had the potential to allow me to investigate host and bacterial diversity and, importantly, provided new insect vector–*Rickettsia* systems to investigate.

The above account highlights the utility of publicly accessible databases not only acting as inspiration for hypothesis generation but also in assisting flailing researchers in realigning a project's trajectory. Correspondingly, the sudden altered culture of working from home as a result of the coronavirus disease 2019 (COVID-19) pandemic resulted in an extra reliance on secondary data analysis for the completion of projects in the absence of lab access [7]. This allowed early career researchers to gain vital data-analytical skills and further exemplifies the merits of research parasitism in the 21st century.

## More is more: The serendipity of storing everything

**Figure 1:**
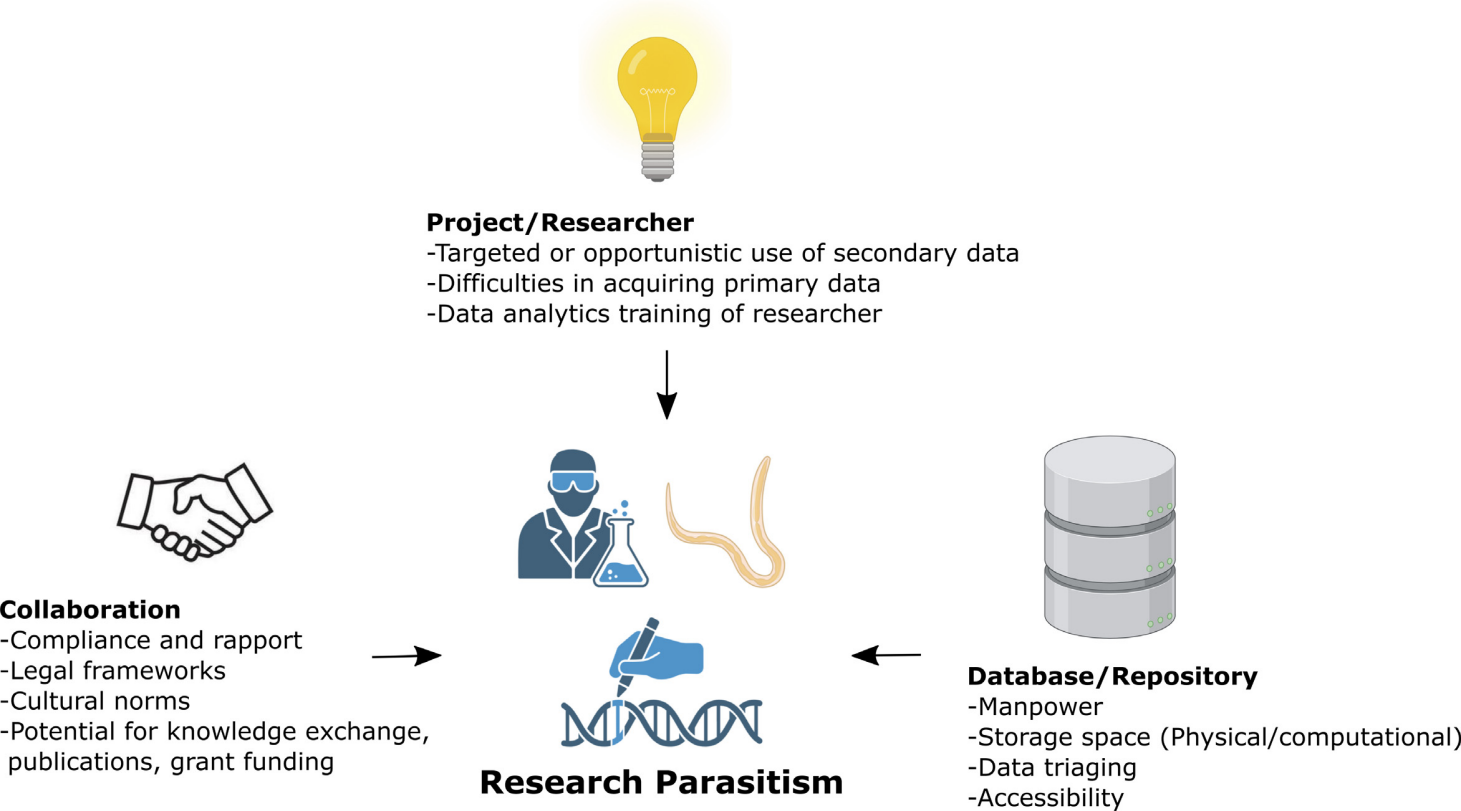
Factors enabling research parasitism.

A difficult process for curators of databases and repositories is the triaging of data, metadata, and samples based on the predicted utility of information for researchers. My recent experience of helping to establish a biobank of severe acute respiratory syndrome coronavirus 2 samples involved cataloguing various tissues, specimens, and swabs from people who developed severe illness as a result of COVID-19. This biobank was constructed in order to offer these samples to a network of collaborators who wished to develop therapeutics, develop vaccines, and understand the manifestation of severe disease beyond respiratory effects. The principal investigators faced the dilemma of which samples to prioritise given limited resources (storage and manpower) and the difficulties of knowing which samples *are and would become* relevant to investigators during the course of the ongoing pandemic and beyond. Similarly, the Centre for Biodiversity Genomics (Guelph, Canada), which hosts BOLD, decided where possible to retain and archive residual DNA from large-scale insect DNA barcoding studies [8]. Despite the additional time, space, and effort taken to create this extra archive, this led to the option of receiving biological samples, which had misamplified *Rickettsia* in order to “re-barcode” and generate the intended DNA barcode of the host. This potential to assist unimagined future projects was anticipated by an investigation intended to understand how to enable global participation in DNA barcoding efforts [9]. Here an anonymous researcher involved with BOLD barcoding studies acknowledged the likelihood of potential research avenues emerging that had not yet been thought of and thus the need to store a much broader set of genetic resources. The same study also illuminated how the collaborating and sharing of resources could be impeded by cultural norms, resource misappropriation, and lack of trust between stakeholders. Thus, although our group was a beneficiary of such a collaboration, to fulfil the potential of DNA barcoding projects globally, there have been suggestions of the need to put in place more stringent governance arrangements and develop awareness of ethical norms as well as the benefits of collegiality within the barcoding community. This idea has been previously identified as being of benefit to global scientific communities wishing to collaborate and make use of the abundance of stored data in an ever more interconnected world sharing challenges and questions (Fig. 1) [10].

Another feature of BOLD lending itself as enabling research parasitism is its way of archiving metadata. Aside from DNA barcode sequences themselves often used to answer the question “What are you?”, selected metadata, such as sample location and photos of the specimen, are stored in order to help understand biodiversity, dispersal events of ancestors, and the detection of cryptic species. *Assumed* prioritised lines of questioning are assisted by the quality control of samples to ensure information is streamlined, reliable, and ultimately of utility to researchers. DNA barcodes submitted to BOLD are screened to ensure they derive from *COI*, there are no stop codons in the middle of the sequence, and the sequence does not derive from a contaminant (e.g., human, parasitoid, bacteria) [6]. Pertaining to the latter, contaminant sequences, instead of being rejected outright, are filtered into a separate database. This was key in allowing me to identify that *Rickettsia* DNA was not only a large proportion of contaminant sequences in barcoding projects but that this untapped information had implications spanning beyond traditional lines of enquiry—namely, the discovery that Torix *Rickettsia* were an underappreciated facet of arthropod biology.

Thus, although curators will have a set of *a priori* questions they think can be answered by storing specific samples, data, and metadata, a culture of storing all information where possible, whether it is deemed on the face of things to be useful or not, can assist in answering those questions not immediately obvious to the scientific community. This enabled me to progress the analysis with colleagues resulting in the paper that was the subject of the research parasite prize. Just like the discoveries of CRISPR-Cas9 and penicillin had unforeseen applications detached from initial motives to study them, the coordination of large data repositories with often perceived redundancies can lead to unexpected innovations and answers to questions that were not conceived of initially during their foundation.

## Data availability

Not applicable

## Abbreviations

BOLD: Barcoding of Life Data System; COI: Cytochrome Oxidase subunit 1; COVID-19: coronavirus disease 2019; CRISPR-Cas9: clustered regularly interspaced short palindromic repeats and CRISPR-associated protein 9; NCBI: National Centre for Biotechnology Information; SRA: Sequence Read Archive.

## Note from the Editors

The 2022 Research Parasite Award was held at the Pacific Symposium on Biocomputing on the Big Island of Hawaii. The establishment of the award was a reaction to an editorial that presented arguments against data sharing, including that it promoted a system where “research parasites” (those who reuse datasets created by “frontline researchers”) would proliferate. As promoters of data sharing, GigaScience Press has each year sponsored the Junior Parasite Award for postdoctoral, graduate, or undergraduate trainees. For more see, the Research Parasite Awards website at https://researchparasite.com/.

## Competing Interests

The author declares no competing interests
